# HealthAge: evaluation of intrinsic capacity changes in humans, mice, and killifish to explore the biology of aging

**DOI:** 10.1007/s11357-025-01718-2

**Published:** 2025-06-16

**Authors:** Sophie Guyonnet, Claudie Hooper, Heike A. Bischoff-Ferrari, Angelo Parini, Yohan Santin, Jean-Philippe Pradère, Cédric Dray, Bruno Vellas

**Affiliations:** 1 IHU HealthAge, Gérontopôle, Department of Geriatrics, CHU Toulouse, Toulouse, France; 2 CERPOP Inserm UMR 1295, University of Toulouse III, Toulouse, France; 3 Department of Aging Medicine, Felix Platter, University of Basel, Basel, Switzerland; 4 I2MC, Institut Des Maladies Métaboliques Et Cardiovasculaires, Inserm UMR 1297, University of Toulouse III, Toulouse, France; 5 Institut RESTORE - UMR 1301 - Inserm/5070-CNRS/EFS, Univ. P. Sabatier, 31037 Toulouse, France

**Keywords:** HealthAge, INSPIRE, ICOPE, Biomarkers, Intrinsic capacity, Aging

## Abstract

HealthAge was devised by a conglomerate of research groups in Toulouse, France, with the combined goal of narrowing the lifespan-healthspan gap through novel translational bench-to-bedside research studies. HealthAge comprises the “INStitute for Prevention” “healthy aging” and “medicine Rejuvenative” (INSPIRE) human translational, outbred SWISS mice and African turquoise killifish (GRZ strain) cohorts in which aging is studied based on the concept of intrinsic capacity (IC). In this narrative review, we describe the three INSPIRE aging models (human cohort, *n* = 1109, age range 20 –102 years old with mean age ± standard deviation, 62.4 ± 19.0 years and 61.9% female; outbred SWISS mice, *n* = 1576 and African Turquoise killifish, *n* = 300) and explain how IC is assessed at the clinical (in humans) and biological level over time. HealthAge strives to elucidate the underlying biology of IC and to identify biomarkers of IC declines and novel gero-therapeutics using the clinical and biological data and biospecimens collected prospectively in the three species. The data sharing policy will foster scientific discovery through new multi-disciplinary collaborations. Thus, HealthAge will promote healthy aging using a unique translational platform based on IC phenotyping with the ultimate goal of preventing loss of human independence and alleviating health costs associated with an aging population.

## Introduction

HealthAge was founded in 2023 and is a large-scale, multi-center collaborative project involving the *Gérontopôle at* Toulouse University Hospital in conjunction with the University of Toulouse and the Institut National de la Santé et de la Recherche Médicale (Inserm) [1]. The main objective of HealthAge is to extend healthspan as human life expectancy has dramatically increased, while healthspan has not followed suit, therefore negatively impacting the quality of life of older adults [2]. In HealthAge, this ambitious goal is tackled by connecting function at the public health level with processes at a biological level centered on the concept of intrinsic capacity (IC) [3].

In 2015, the World Health Organization (WHO) published the first World Report on Ageing and Health [3] in order to implement a new public-health strategy on aging. In this report, the WHO defined healthy aging as “*the process of developing and maintaining the functional ability that enables people to be and to do what they have reason to value*.” Functional ability is determined by the IC of an individual, which is the composite of all the physical and mental capacities plus interacting environmental influences [2–4]. To operationalize IC in a clinical context, the WHO have identified six connected core components reflecting multiple physiological functions relevant to aging: *locomotion, cognition, vitality, vision, audition*, and *psychological well-being*. Evidence demonstrates that deficits in IC strongly predict adverse health outcomes including falls, care dependency, disability, and mortality [6]. IC and its six domains can be assessed clinically according to the recommendations in the WHO Integrated Care for Older People (ICOPE) handbook (5 step process) [7]. This can be achieved through the use of an application (ICOPE Monitor that was developed and trialed at the *Gérontopôle*, Toulouse University, France), a web platform or through a telephone call with a trained research nurse [8, 9]. ICOPE represents an innovative, patient-centered system to manage and optimize health and well-being. Note this review is founded on the original ICOPE program for human IC assessment; however, ICOPE 2 (4 step process) has recently been released by the WHO, which represents a refined version with expanded assessments and clearer pathways for its integration into primary care [10].

As healthcare systems face the growing burden of aging populations, ICOPE provides a structured framework that combines preventive care, clinical management and social support services. This holistic approach aims to enhance health outcomes through long-term follow-up, reducing hospitalizations, long-term care dependency and overall healthcare costs. Implementing ICOPE in primary care will generate valuable insights into aging trajectories, enabling researchers to identify patterns, assess intervention efficacy and refine strategies for extending healthspan. This new shift in the health paradigm will in turn advance the field of geroscience through the promotion of healthy aging. Recently, a large-scale feasibility study performed at the *Gérontopôle*, Toulouse University Hospital (France) has provided evidence to support the implementation of ICOPE on a large scale in clinical practice [8, 11]. Through the use of the ICOPE program, the WHO aims to reduce the number of dependent people by 15 million worldwide by 2025 [12].

The main purpose of this narrative review is to describe the unique HealthAge initiative encompassing the human, outbred SWISS mice and African Turquoise killifish (TK) cohorts and describe how IC is measured longitudinally in each species. The animal models with their accelerated aging profiles in conjunction with human phenotyping will expedite discovery and validation of novel biomarkers and drug targets that protect against IC declines.

### The INSPIRE-T human cohort

The INSPIRE translational (INSPIRE-T) human cohort is a 10-year, observational, repeated measures study, designed to capture aging trajectories based on the WHO ICOPE (original version) program and the assessment of IC. The INSPIRE-T study was approved by the French Ethics Committee located in Rennes (CPP Ouest V) and the study was registered at http://clinicaltrials.gov (NCT04224038). Recruitment to the study began in October 2019 and all participants gave signed informed consent with the target number of subjects (*n* = 1000) being recruited by December 2021. The open cohort (ongoing recruitment) consists of 1109 participants with data available at baseline (61.9% female) aged from 20 to 102 years old (mean age ± SD, 62.4 ± 19.0 years) recruited from the Toulouse area (France) exhibiting varying levels of functional status. Sample size calculations were not performed due to the exploratory nature of HealthAge. We considered an approach based on the potential to obtain parameter estimates with sufficient precision informed by 1000 human subjects, which represents the maximum number of people that can be monitored with the available funding. The same rationale was used to derive the numbers of mice (*n* = 1576) and TK (*n* = 300). Participants in the INSPIRE-T cohort are stratified into 10-year age groups with oversampling of participants ≥ 70 years old to compensate for increased drop-out in this age bracket and to enable the better investigation of frailty and age-related disease. Recruitment to the study remains permanently open to maintain a minimum pool of 1000 participants. The rationale, methodology, and objectives of the INSPIRE-T study are comprehensively reviewed elsewhere [12, 13].

In brief, data collection in the INSPIRE-T human cohort consists of information on the six domains of IC assessed using the ICOPE program. Participants had IC (ICOPE Step 1) monitored every 4 months during the first year of the study then every 6 months for the remainder of the study. Participants undergo an additional thorough clinical assessment (ICOPE Step 2) and blood sampling if IC declines are detected, the latter enabling the investigation of blood-borne mediators expressed at the same time that IC declines are apparent. Demographic measures, information on health status, cognition (Mini Mental State Examination, MMSE [14]), Fried’s frailty phenotype [15], functional ability [16, 17], oral health [18], lifestyle (including physical activity, sleep statistics, diet [19, 20], smoking status and alcohol consumption), as well as participant-reported outcomes (cognition [21], mobility, fatigue, social isolation [22]) are also collected to characterize participants and provide supplementary information on IC domains. Participants will be evaluated once a year for all clinical assessments throughout the 10-year study. Table 1 (column 1) provides a full list of clinical tests that are performed in the INSPIRE-T human cohort in order to capture IC.

**Table 1:** A comparison of screening tests for intrinsic capacity in humans, outbred SWISS mice, and turquoise killifish in HealthAge

IC Domains	Tests used in humans to assess intrinsic capacity (ongoing screening)	Tests used in the outbred SWISS mice to assess intrinsic capacity	Tests used in the turquoise killifish to assess intrinsic capacity
Locomotion	**ICOPE Step 1 test**: Mobility is measured by the time (in seconds) required to perform 5 chair rises at a maximum speed. Declines are evident when the time needed to complete the test is greater than 14 s for subjects < 80 years or greater than 16 s for subjects ≥ 80 years. **Other tests used in HealthAge to probe the locomotion domain**: -Short Physical Performance Battery (SPPB) (score/12) with lower scores indicative of worse locomotion (ICOPE Step 2 test). -Chair rise test (number of rises in 30 s). -PROMIS: mobility (T-scores^*^). -Isokinetic muscle strength (Cybex).	-The “open-field test” was used to study locomotor activity (walking speed). Mice were positioned in the central area of a polyvinyl chloride cylinder (40 cm diameter) for a duration of 10 min. Walking speed was recorded using EthoVision software. - The “Incremental treadmill test” was used to assess running speed and time. The test protocol included a warm-up phase at 6 m/min for 6 min followed by a continuous increment of 1 m/min every minute.	-Spontaneous and forced (using a swim tunnel) locomotion was recorded by video tracking (24/7) using EthoVision software for analysis. Metrics recorded from 10-min video extracts included distance moved, average speed and maximum speed.
Psychological well-being	**ICOPE Step 1 test**: Psychological well-being/depressive symptoms are measured by the following two questions: Over the past 2 weeks have you been bothered by: 1. Feeling down, depressed or hopeless? 2. Having little interest or pleasure in doing things? A «yes» response determines a decline. **Other tests used in HealthAge to probe the psychological well-being domain**: -Patient Health Questionnaire-9 (PHQ-9) (score/27) with higher scores indicative of a higher risk of depression (ICOPE Step 2 test).	-The “open-field test” was used to assess anxiety-like behaviors such as reluctance to explore the centre of the arena and increased thigmotaxis/wall following. The same arena was used as for locomotor assessment. The percentage of time spent in the centre of the arena was recorded using EthoVision software.	-Swimming patterns were recorded by video tracking (24/7) to evaluate anxiety (novel tank diving assay), aggressiveness and temerity using EthoVision software for analysis. In the novel tank diving assay, an increase in over-time vertical swimming from the initial position at the bottom of the tank is interpreted as a reduction in anxiety.
Cognition	**ICOPE Step 1 test**: Cognition is measured by the 3-word remember test and the following four orientation questions: Which year is it? What month is it? What day of the month is it? What day of the week is it? from the MMSE. An abnormality was recorded if an individual was unable to remember at least one word or if they provided an incorrect response to an orientation question. **Other tests used in HealthAge to probe the cognition domain:** -MMSE (score/30) (ICOPE Step 2 test). -For participants < 70 years old the free and total recall of the Free and Cued Selective Reminding Test (FCSRT: score/96), ten MMSE orientation items (score/10), the Digit Symbol Substitution Test (DSST) from the Wechsler Adult Intelligence Scale—Revised (symbols/90 s), and the Category Naming Test (CNT) (2-min category fluency in animals) -Cognitive Function Instrument (CFI) (score/14). Lower scores are indicative of worse cognition for all cognitive tests apart from the CFI. -Activities of Daily Living (ADL) (overlap with locomotion domain). -Instrumental Activities of Daily Living (IADL) (overlap with locomotion domain). ADL and IADL: higher scores indicate greater functional independence. -Brain MRI.	-Y-maze spontaneous alternation test was used to assess spatial working memory. Mice were placed in one of three arms of a Y shaped maze and allowed 8 min for exploration. The percentage of alternation, derived from the ratio of alternations to total arm entries served as the measure of memory function.	-X-maze and T maze tests in conjunction with a Pavlovian conditioning model were used to test neophobic behavior, associative memory (with a reward or stressor) and learning abilities (repetitive tasks). Cognition was assessed in young (2 months of age) versus old fish (5–6 months of age). Experiments were recorded by video tracking using EthoVision software for analysis.
Vitality	**ICOPE Step 1 test**: Vitality/malnutrition is measured by the following two questions: Have you unintentionally lost more than 3 kg over the last 3 months? Have you experienced loss of appetite? A «yes» response determines a decline. **Other tests used in HealthAge to probe the vitality domain:** -Mini Nutritional Assessment (MNA) (score/30) with higher scores indicative of better nutrition (ICOPE Step 2 test). -Body Mass Index. -Food Frequency Questionnaire (FFQ). -Oral status assessed using the Oral Health Assessment Tool (OHAT) with higher scores indicative of worse oral health. -PROMIS: fatigue and social isolation (T-scores^*^). -Body composition (Dual-energy X-ray absorptiometry, DXA). -Whole body MRI. -Maximum oxygen consumption (VO_2_ max).	-Limb grip strength was used to measure the force exerted by the mouse forelimbs. Mice were positioned above a horizontally placed grid and drawn along a straight line leading away from the sensor. Peak force was identified as the highest value obtained from 5 trials for each attempt and attempts were conducted three times at hourly intervals. -Tight-rope test was used to assess motor coordination in the mice. Mice were positioned in the centre of a 60 cm tightrope (2 mm diameter) suspended 70 cm from the floor. Success in the test was defined by mice reaching one of the cable ends or maintaining balance on the tightrope for the maximum test duration of 240 s. -Weight change over 1 month was assessed as a percentage. Mice were weighed at 8 am ± 30 min to mitigate variations caused by food intake and activity. -Cardiac function was assessed using standard 2D echocardiographic examination on anaesthetized mice. Examination was conducted in the parasternal long-axis view to evaluate left ventricular dimensions and systolic function and short-axis view to assess chamber dimensions, wall thickness and fractional shortening. To assess diastolic function, transmitted pulsed wave Doppler echocardiography was performed. The E/A ratio was calculated and isovolumetric relaxation time. -Urinary function was assessed by void spot assay. Mice were placed in a standard cage with the floor covered with absorbent paper for 2 h. Following the return of the mice to the home cage the filter paper was dried and exposed to UV light to visualize urine spots. Urine spots, total urine area and voiding patterns were evaluated. - Complete blood counts were performed using a ProCyte Dx analyzer.	-Growth (weight and length) was measured every 2 weeks throughout life-course. -Metabolic blood parameters including glucose and lactate were measured in 10 µl of blood using an Accu-check glucometer (Roche) or the Lactate Pro 2 reader (Arkray) respectively. Metabolic parameters were measured in young (2 months of age) versus old fish (5–6 months of age). -Measurement of reaction time for fish to reach food at the water surface (in seconds) and determination of food intake using fluorescent artemias. -Mitochondrial morphology and ultra-structure were assessed in skeletal muscle by transmission electron microscopy to assess metabolic changes that could potentially influence vitality. Ultrathin Sects. (70 nm) were fixed in Sorensen phosphate buffer and stained with 3% uranyl acetate then observed using a HT 7700 Hitachi transmission electron microscope at an accelerating voltage of 80 kV. Mitochondrial morphology was assessed in young (2 months of age) versus old fish (5–6 months of age).
Audition	**ICOPE Step 1 test**: Audition is measured using the whisper test. The evaluator softly whispers a common two-syllable word and asks the person to repeat the word. The evaluator then tests the other ear using a different word. Not repeating the correct words determines a decline. If the whisper test cannot be performed then two questions are asked: Did you notice a worsening of your hearing in the last 4 months or since the last evaluation? Does your family complain of an acute recent hearing loss? -Screening audiometry with tones across the speech spectrum (500 to 4,000 Hz) at the upper limits of normal hearing. Results are recorded as pass or abnormality/refer. A reading of 35 dB or less indicates normal hearing (from year 1 of the study).	Not measured	- Not measured
Vision	**ICOPE Step 1 test**: Vision is measured by the following questions: Do you have any problems with your eyes: difficulties in seeing far, reading, eye diseases or currently under medical treatment? Declines are considered present when a person responds «yes» to this question if they are not under regular ophthalmological care. **Other tests used in HealthAge to probe the vision domain:** -WHO simple eye chart for near and far vision (deficit: yes or no) (ICOPE Step 2 test). -Amsler Grid test for detection of age-related macular degeneration (macular degeneration: yes or no).	Not measured	Not measured

*ADL *activities of daily living; *CFI *Cognitive Function Instrument; *CNT *Category Naming Test; *DSST *Digit Symbol Substitution Test; *DXA *dual-energy X-ray absorptiometry; *FCSRT*, the Free and Cued Selective Reminding Test; *FFQ*, Food Frequency Questionnaire; *IADL*, Instrumental Activities of Daily Living; *MMSE*, Mini Mental State Examination; *MNA*, Mini Nutritional Assessment; *MRI*, magnetic resonance imaging; *OHAT*, Oral Health Assessment Tool; *PHQ-9*, Patient Health Questionnaire-9; *PROMIS*, Patient-Reported Outcomes Measurement Information System; *SPPB*, Short Physical Performance Battery; *WHO*, World Health Organization

^*^T-scores are standardized scores with a mean of 50 and a standard deviation (SD) of 10 (interpretation: scores < 40 are more than 1 SD below average and scores > 60 are more than 1 SD above average)

To further probe the biology underlying IC, all participants underwent dual-energy X-ray absorptiometry (DXA) measurements for body composition at baseline and year 2 (with subsequent 5 yearly follow-ups planned). Whole body and brain magnetic resonance imaging (MRI) was also performed at study baseline in a subset of participants (*n* = 113). Furthermore, maximum oxygen consumption (VO_2_ max) as well as isokinetic muscle strength (Cybex) was measured between baseline and 24 months in a subset of participants (*n* = 279 and *n* = 286 respectively).

A biobank was established from biospecimens including blood, urine, saliva, and dental plaque, which were collected at study baseline, then annually as detailed elsewhere [12]. Nasopharyngeal swabs, stools, hair follicles, skin surface samples, and skin biopsies were also collected optionally at baseline (stool and hair follicle samples will be subsequently collected every 2 years throughout the study).

### The INSPIRE mouse cohort

The INSPIRE mouse cohort consists of 1576 outbred SWISS mice (Janvier Labs, France) and is described in detail elsewhere [23, 24]. Outbred SWISS mice were chosen as a model system as they retain substantial genetic variability in comparison to the more traditional inbred laboratory mouse strains and thereby provide a more suitable murine model to reflect the diversity of human genetics. The colony consists of both male and female mice enabling inter-sex differences to be explored throughout the course of aging. The mice were housed 4 per cage in Digital Ventilated Cages (DVC®) (Tecniplast, Italy) in a temperature-controlled environment (21 °C ± 2 °C) with a 12-h light–dark cycle and had unrestricted access to food and water. This caging system enables 24 h/7 d data collection on locomotion, body weight, as well as food and water consumption.

The mice were divided into four groups to better model human habits: control animals, high fat/high sucrose diet fed (HF/HSD), “wheel” (to enable voluntary physical activity) and HF/HSD diet + wheel. This experimental paradigm mimics “healthy” versus “unhealthy” human lifestyles focusing on nutrition and exercise as the lifestyle variables. Subgroups of mice from these four groups were culled at 6, 12, 18, and 24 months, and these mice were designated the “cross-sectional cohort” (*n* = 1456) [23, 24]. These time points correspond to an approximate age range of 30 – 80 years old in humans. The remaining 120 mice (60 females and 60 males) were designated the “longitudinal cohort” and were left to live a natural lifespan solely to determine strain longevity under our particular housing conditions (without intervention or assessment). Of note, the mice in the longitudinal cohort were under control feeding conditions.

Key components of IC were assessed in the cross-sectional mouse cohort at 6, 12, 18, and 24 months as an approximation to ICOPE vitality, locomotion, cognition, and psychological well-being screening. Vision and audition were not assessed in the mouse cohort at this time. In a future planned mouse aging study, vision and audition will be monitored and evaluated using a novel digitalized system that is currently under development. Thus, the four aforementioned IC domains were assessed through a non-invasive “IC score,” an adapted version of the previously described Valencia Frailty Score for mice [25]. This score includes as measures of vitality: % body weight change (over 1 month), grip strength, and motor coordination assessed using the tight-rope test. Locomotion was also measured through the assessment of spontaneous walking speed using the open-field test and running speed (and time) using the incremental treadmill test [24]. To better mirror human ICOPE IC screening, cognition (working memory) was evaluated using the Y-Maze spontaneous alternation test [26], and as a measure of psychological well-being, the open-field test was performed to assess anxiety [27]. The same open-field test as for locomotion was used where anxiety-like behaviors such as reluctance to explore the center of the arena and increased thigmotaxis (wall following) were observed in an unfamiliar open space.

Cardiorespiratory function has been suggested to indirectly influence the vitality domain of IC through metabolic changes [5], thus cardiac systolic and diastolic functions were examined by echocardiography and Doppler echocardiography at 6, 12, 18, and 24 months in the mice. Urinary function was also measured at the same time points using a spontaneous void spot assay due to the association of urinary incontinence with social hindrance and loneliness in humans [23, 24]. Social isolation and loneliness have in turn both been associated with worse cognition and Alzheimer’s disease in older adults [28]. Indeed, ICOPE 2 now includes clinical assessment and support for people with urinary incontinence [10]. Furthermore, complete blood counts were conducted in the mice at 6, 12, 18, and 24 months to assess hematological factors including red blood cells, reticulocytes, and platelets as well as myeloid and lymphoid cell populations [24]. At the time of endpoint euthanasia of the mice subgroups, blood, feces, urine, and organs were collected and stored for future biomarker studies.

### The INSPIRE turquoise killifish cohort

The African Turquoise Killifish (TK) (*Nothobranchius furzeri*) are short-lived vertebrates with a median lifespan of 3–8 months, which therefore lend themselves to aging studies [29]. The rapid aging of TK shares several characteristics with human aging including telomere shortening, cancer, immuno-compromisation, reduced locomotion and cognitive decline [29]. The INSPIRE TK cohort (GRZ strain) consists of a replenished, ongoing cohort of about 300 TK with an approximate 50:50 ratio of males to females, thus enabling inter-sex differences in aging to be explored as with the SWISS mice cohort. The TK were raised at 28 °C in a central filtration recirculating system with a 14-h light/10-h dark cycle under standard laboratory conditions. The fish were housed individually, with one fish per 3 L of system water. The influences of lifestyle factors (diet and exercise) on IC were not assessed as a function of aging as with the mice cohort. All TK were left to live a natural lifespan, and in the main cohort, noninvasive IC measures of vitality, locomotion, and cognition were taken over time.

To provide measures of vitality, in a sub-population of TK (*n* = 30), growth (weight and length) was measured every 2 weeks throughout lifespan. Furthermore, metabolic blood parameters including glucose and lactate levels were measured in young TK (2 months) versus old TK (5–6 months). Vitality was also determined by measuring the reaction time for fish to reach food at the water surface, and food intake was assessed using fluorescent artemias (shrimp) through the subsequent detection of the fluorescent signal in the gut and feces.

Locomotion both spontaneous and forced (using a swim tunnel) as well as other behaviors including aggressiveness and temerity were investigated using a 24 h/7 d infra-red video tracking system. Anxiety was evaluated using the Novel tank diving assay where an increase in over-time vertical swimming from the initial position at the bottom of the tank is interpreted as a reduction in anxiety [30]. Collectively, anxiety, aggressiveness, and temerity provide measures of fish psychological well-being. At present, recorded swimming patterns are under analysis using computer-assisted methods in collaboration with the Computing Research Institute in Toulouse (Institut de Recherche en Informatique de Toulouse). Further pertaining to the locomotion domain of IC, the cross-sectional area of the fish trunk was measured to monitor muscle loss with age as a model of sarcopenia in the fish.

Cognition in young TK (2 months) versus old TK (5–6 months) was assessed using X and T maze set-ups using a Pavlovian conditioning model to provoke swimming [31]. Mitochondrial morphology was also examined in skeletal muscle of young versus old TK (2 months versus 5–6 months) using electron microscopy providing a measure of age-related metabolic changes, which have been implicated in the vitality domain of IC [5]. To date, vision and audition have not been assessed in the TK. However, vision will be assessed using an optokinetic test chamber [32], and audition will be investigated by studying the auditory-evoked startle response and hearing sensitivity to acoustic startle stimuli in future experiments [33]. Additionally, histo-morphological analysis of the retina will be performed in both the larvae and adult fish to examine structural changes in the eye occurring with age. Complete blood counts will also be performed in young versus old TK from samples isolated from the head kidney (playing a similar role to the bone marrow in humans), blood, and gills to mirror the hematological assessments performed in the mice [24]. In contrast to the INSPIRE mouse cohort, organs and blood samples were not routinely collected and stored due to the lack of biological matter yielded per TK for subsequent laboratory analysis. Instead, biomarker studies will be conducted on tissue from whole TK.

### Common experiments across species for the identification of biomarkers of IC declines

Both targeted and untargeted (discovery-based) techniques will be employed to identify novel biomarkers of IC declines per se as well as biomarkers of declines in its constituent domains using the biological specimens collected from all three species. In line with the expertise available in the HealthAge research community, the following bio-processes will be examined: organ function, cognition, immune-function/inflammation, metabolism and mitochondrial biology. Specific targets to be investigated include the following: ATPase inhibitory factor 1 (IF1), T-cell subset composition, fat distribution, and apelin. The reasons for this are that these pleiotropic mediators play a role in physiological processes that are altered in disease and are thereby intimately linked to IC. *ATPase inhibitory factor 1 (IF1)* is an endogenous inhibitor of mitochondrial ATP synthase (of the electron transport chain) and plays a pathological role in disease states such as cancer, cardiovascular disease, and diabetes [34]. Altered *T-cell subset distribution* contributes to immuno-senescence leading to increased susceptibility to a multitude of age-associated diseases [35] and increasing evidence suggests that *fat distribution* in different depots affects overall health and particularly metabolic health [36]. Lastly, *apelin*, a putative biomarker of sarcopenia [37], is of interest because it is involved in many biological processes including cardiovascular homeostasis, neuroprotection, immunity, oxidative stress and metabolic disorders [38, 39]. The multiple functions of apelin are attributed to the wide expression pattern of its receptor, APJ [38, 39].

In terms of the untargeted biomarker discovery research arm, multi-omics including transcriptomic, metabolomic, lipidomic, proteomic as well as whole genome epigenetic studies will be performed on the collected biospecimens. Multi-omics will be used to identify the most abundantly changing targets to produce ranked lists in the three species for comparative analysis. We will subsequently use linear mixed effect models to investigate whether single biomarkers associate with IC declines over time. Receiver Operating Characteristic (ROC) curve and Area Under Curve (AUC) analyses will also be used to assess biomarker performance. Machine learning algorithms will be employed to analyze the ability of multiple biomarkers to predict IC declines where interactions and more complex non-linear relationships might be at play. The tissues/organs obtained from the animals provide a valuable resource for biomarker identification as such parallel tissues are not available in humans in HealthAge. Nevertheless, putative biomarkers of IC declines will be cross-validated where possible in humans. Furthermore, the causal role of biomarkers in loss of IC will be explored where mechanistically relevant using the animal models in order to identify potential gero-therapeutic targets for the prevention of IC declines in humans.

## Discussion

Here we describe the HealthAge initiative integrating IC phenotyping in the INSPIRE human translational, outbred SWISS mouse and TK cohorts (Table 1). Using these three unique models and the biospecimens collected, IC trajectories across species will be captured and the underlying biology will be studied at the clinical, tissue, cellular, and molecular levels. We also plan to explore IC trajectories as a function of lifestyle factors including nutrition, physical activity/sedentary behavior as well as sleep and social isolation in the human study. Considering that lifestyle modifications are easy to implement and low-cost compared to drug therapies, they represent a promising avenue for the maintenance of IC. Indeed, a growing body of evidence suggests that lifestyle and psychosocial factors are important determinants of IC [40].

Many large studies of aging are in existence and include the Baltimore Longitudinal Study of Aging [41], the Canadian Longitudinal Study on Aging (CLSA) [42], and the English Longitudinal Study of Ageing (ELSA) [43] to name but a few. The human INSPIRE-T study adds to these studies and is novel in design through its specific capture of IC and its six domains temporally offering a more global and comprehensive picture of the aging process. Furthermore, to the best of our knowledge, HealthAge represents the first study to specifically parallel data capture on IC in three species in a concerted effort to rapidly advance geroscience research with the objective to prolong healthspan. This is achievable since many features of aging are conserved across species [44, 45]. Using our three-armed model, HealthAge aims to identify novel biomarkers of adverse IC trajectories using both targeted research and untargeted multi-omic studies. Furthermore, using the ensemble of biomarkers identified and following validation in humans, we plan to develop a novel IC clock that will enable the clinical assessment of biological versus chronological IC status (potentially reflecting age acceleration/deviation) for the better prediction of IC changes. Our IC clock will complement the existing aging clocks that generally only employ one ‘omic’ method such as the Horvath [46] and Hannum clocks [47], which are both based on epigenetic changes. Only a few clocks have employed multiple biomarkers for tracking biological age [48, 49]. The advantage of using multiple biomarkers is that it captures more diverse changes in the many mechanistic drivers of aging. Classical, well-accepted drivers of aging in the field of geroscience include chronic low-grade inflammation, altered adaptation to stress, metabolic changes, free-radical induced macromolecular damage and telomere shortening, de-regulated proteostasis, stem cell changes, and cell senescence, as well as epigenetic modifications [50, 51] with others yet to be identified. We hope to capture signatures of many of these processes with our multi-omic studies.

**Fig. 1:**
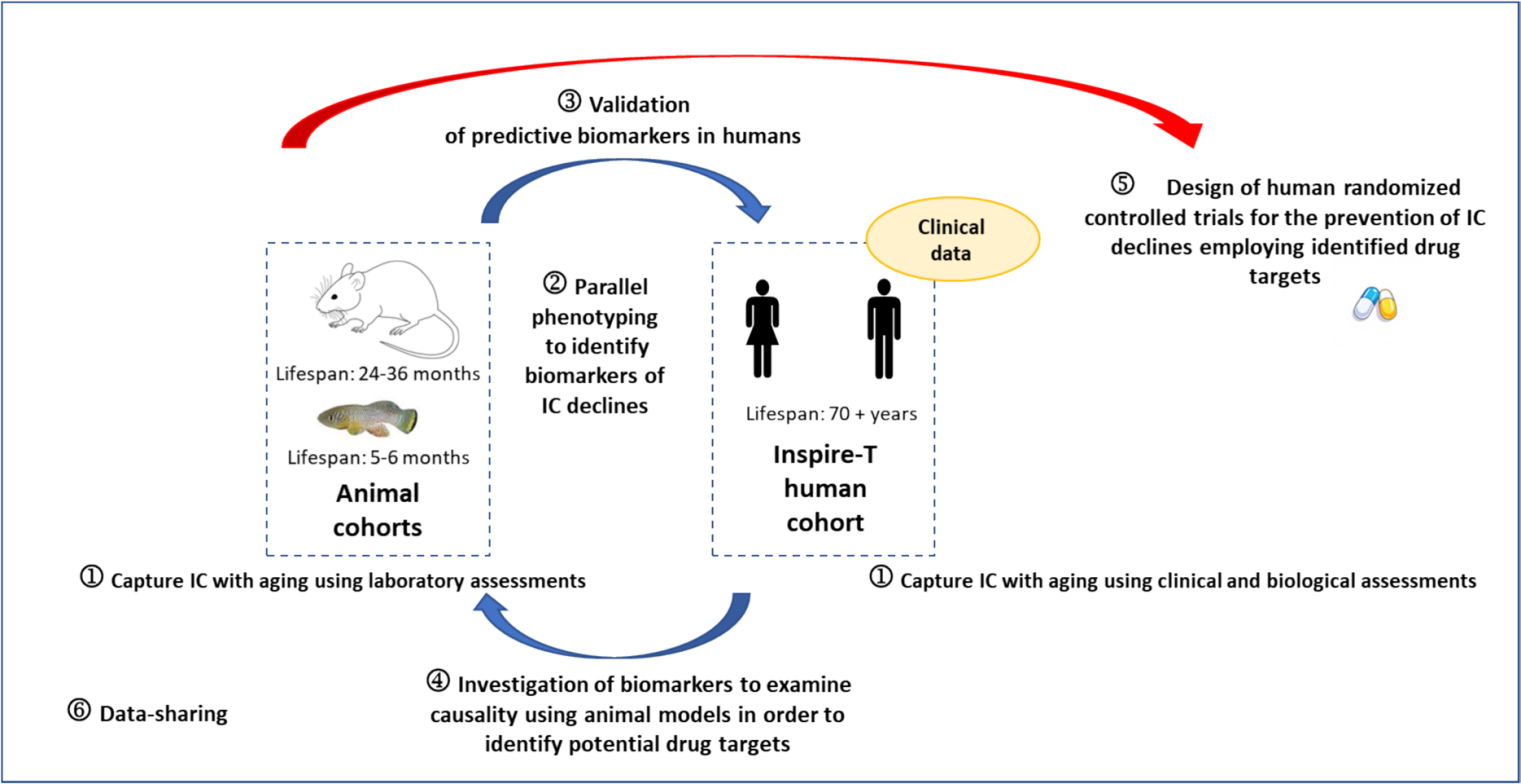
The HealthAge pipeline to identify biomarkers of IC declines and determine causality of relevant biomarkers for the design of human drug trials. IC trajectories will be captured using clinical and biological assessments in humans and laboratory assessments in animals. Biomarkers of IC declines will be identified using the biospecimens and biological data collected from the cohorts. Animals with their short lifespan will expedite biomarker discovery. The causal role of biomarkers will be explored where mechanistically relevant using the animal models in order to identify drug targets for the prevention of IC declines. Randomised clinical trials employing identified targets will then be performed in humans to assess clinical efficacy of potential gero-therapeutic agents

Another goal of HealthAge is to identify novel gero-therapeutics (either protein, nucleic acid or small molecule-based both new or repurposed) for the prevention of IC declines and thus the optimization of global function. The animal models, particularly TK, with their accelerated aging profile, provide ideal models for rapid discovery, the results of which will be translated to humans through the implementation of trials for the prevention of IC declines (Fig. 1). By bridging the gap between preclinical and clinical research, HealthAge has the potential to reshape geroscience, leading to interventions that extend healthspan and improve the quality of life in aging populations.

The strengths of the HealthAge initiative include the 10-year follow-up of the human cohort, the in-depth assessments and diverse biological material collection, and the ability to expediate the identification of novel biomarkers of IC in animals for subsequent validation in humans. We have also developed a more comprehensive index of IC scoring in mice to better parallel human studies. A great strength of the study is its commitment to data, knowledge, and specimen sharing, which will enable new collaborations to be forged in turn accelerating geroscience research. The limitations of the study include the fact that the human study sample is not representative of the general population in terms of the high level of education possessed by the participants (71.9% educated at university level) and the under-representation of subjects with low socio-economic status. Recruitment strategies are currently under consideration to address this bias. Furthermore, laboratory light–dark cycles, temperature control, and animal-housing conditions do not fully replicate human living environments.

In conclusion, we describe here the unique HealthAge initiative integrating the INSPIRE human translational, outbred SWISS mice and TK cohorts. This platform will help provide a better understanding of the aging process and the spectrum of IC trajectories. This is in accordance with the WHO’s first World report on Ageing and Health that has reconceptualized healthy aging based on the framework of IC [3]. Accomplishing our objectives in HealthAge will also enable the identification of novel biomarkers and drug targets, which will help predict and prevent IC declines thereby closing the healthspan-lifespan gap. This in turn will reduce care dependency and the utilization of healthcare services conferring significant beneficial economic impact. Thus, HealthAge potentially represents a significant milestone in the advancement of the field of geroscience.
